# Mitochondrial ribosomes in apicomplexan and trypanosomatid parasites: Dissimilar drivers of complexity and convergent features

**DOI:** 10.1371/journal.ppat.1013920

**Published:** 2026-02-09

**Authors:** Ondřej Gahura, Prashant Chauhan

**Affiliations:** 1 Institute of Parasitology, Biology Centre, Czech Academy of Sciences, České Budějovice, Czech Republic; 2 Faculty of Science, University of South Bohemia, České Budějovice, Czech Republic; Institute of Parasitology, Biology Centre, Czech Academy of Sciences, CZECHIA

## Introduction

Mitochondrial ribosomes (mitoribosomes) are responsible for protein synthesis in mitochondria, endosymbiotic organelles with own genomes typically encoding a handful of mostly membrane proteins. Unlike relatively uniform eukaryotic cytosolic ribosomes, mitoribosomes are remarkably diverse, as revealed by cryo-electron microscopy (cryoEM), which documented compositional and structural differences across lineages [1–6]. Generally, mitoribosomes are characterized by lineage-specific gains and losses of proteins, as well as alterations in RNA content, most commonly—but not exclusively—its reduction. These differences contribute to architectural variability with implications for mechanisms of mRNA recognition, translation cycle, or biogenesis. Mitoribosomes that deviated to the largest extent from their bacterial ancestors are found in parasites from two unrelated groups: trypanosomatids (supergroup Discoba), represented by structures from *Trypanosoma brucei* [7], *T. cruzi,* and *Leishmania* [8], and apicomplexans (supergroup Alveolata), represented by structures of mitoribosomes from *Toxoplasma gondii* [9,10]. The two ribosomes stand out for several unparalleled features or extreme cases of other atypical properties. They display attributes that are common to both yet have arisen independently. Driving forces and biological context of the evolution of the convergent properties may not necessarily be the same in both groups.

## What makes *Toxoplasma* and trypanosome mitoribosomes extraordinary?

**Fig 1:**
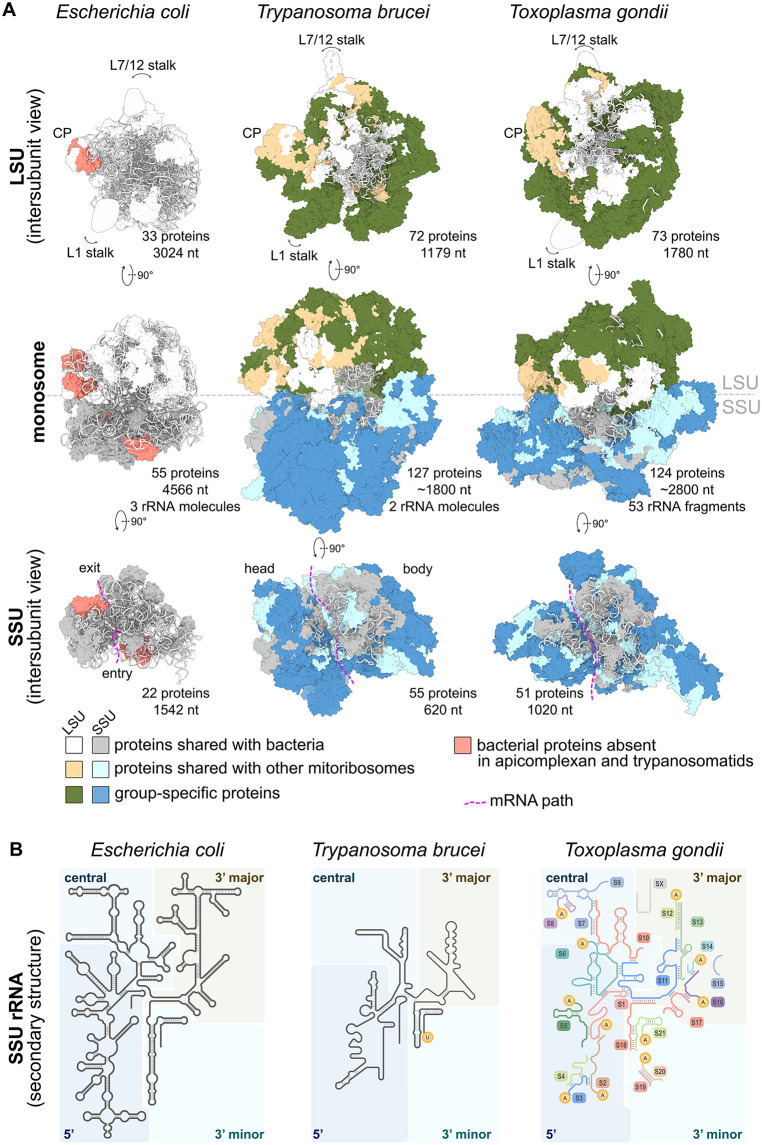
Architectural and compositional overview of apicomplexan and trypanosomatid mitoribosomes. **(A)** CryoEM structures of *Toxoplasma gondii* (PDB IDs 9HQV and 9I05 [10]) and *Trypanosoma brucei* (PDB IDs 6HIV [7] and 7ANE [8]) mitoribosomes and their subunit compared to structure of bacterial (*Escherichia coli*, PDB ID 7K00 [11]) ribosome. Ancestral and group-specific proteins are color-coded, number of proteins and rRNA molecules are indicated. **(B)** Secondary structures of rRNA from small (mito)ribosomal subunits from *T. brucei*, *T. gondii*, and *E. coli*. In *T. gondii*, individual rRNA fragments are showed in different colors and numbered. PolyA and polyU tails are indicated by respective symbols.

In line with their ancient pre-LUCA origin and central role in the catalytic process, the functional cores of the large and small subunits (mtLSU and mtSSU) of trypanosomatid and apicomplexan mitoribosomes responsible for peptidyl bond synthesis and mRNA decoding, respectively, resemble to those of all other ribosomes [7,10]. Other otherwise generally conserved parts, including intersubunit interface, exit and entry of mRNA channel, or bases of flexible mtLSU stalks, which mediate interactions with elongation factors, have deviated markedly from the ancestral bacterial-like state or from more plesiomorphic mitoribosomes, found for example in plants [5]. The least conserved are the outer shells of both subunits, which are composed mostly of lineage-specific proteins or extensions of proteins of bacterial origin. Therefore, visually apparent departures from the typical ribosomal morphology, such as the conspicuously large mtSSU, which is bigger than the mtLSU in both groups, are attributed almost exclusively to proteinaceous components (Fig 1A). However, it is presumably the RNA content that underlies the profound divergence, albeit in different ways in the two groups. Total amount of RNA in trypanosomal mitoribosomes (1799 nucleotides) is the lowest of the structurally characterized ribosomes and not much higher than in mitoribosomes of diplonemids, related marine protists with the most reduced ribosomal RNA (rRNA) documented experimentally (1304 nt in *Diplonema papillatum*) [12]. Trypanosomal mitoribosomal RNA is not only reduced, but also extremely AU-rich, with only 16.6% of GC content. Consequently, many double-stranded regions (helices and stem-loops) present in bacterial ribosomes or other mitoribosomes are either completely lost or reduced to anti-parallel RNA strands without regular base-pairing interactions (Fig 1B). Despite loss of several secondary structure elements, mitoribosomal RNA content in *Toxoplasma* is not exceptionally low (2800 nt). However, its rRNA is fragmented into multiple pieces ranging from 7 to 278 nt. There are other reported cases of RNA fragmentation in cytosolic [13] and mitochondrial ribosomes [3,4,6], including cytosolic ribosomes of trypanosomatids [14–16]. Whereas fragmentation of cytosolic rRNA arises during post-transcriptional processing of pre-rRNA precursors, mitochondrial rRNA fragments are encoded as separate units within the mitochondrial genome. In either case, the fragmentation in *T. gondii* with 21 and 32 pieces in mtSSU (Fig 1B) and mtLSU, respectively, is by far the most pronounced. Uniquely, nine fragments occur in the structure in two copies. Due to the complex architecture of the *T. gondii* mitochondrial genome, each copy contains a specific extension (see reference [12] for details). In all cases, the shared part is folded in an ancestral structure in one copy whereas in the second copy, it adopts an alternative conformation in a different position, contiguously to an ancestral fold of the copy-specific extension [10].

## What do *Toxoplasma* and trypanosome mitoribosomes have in common?

The two different departures from the canonical rRNA scheme, i.e., reduction in trypanosomatids and fragmentation in apicomplexans, are associated with conceptually similar structural modifications. Both require extrinsic stabilization by proteins. In trypanosomes, proteins structurally replace some of the missing rRNA secondary elements and provide scaffold for vestigial single-stranded rRNA stretches or originally double-stranded regions that have lost ability to base pair [7]. In *Toxoplasma*, proteins are vital for stable association or conformational stability of rRNA fragments, especially the short ones [10]. Structurally, the stabilization is achieved by stacking of aromatic amino acid residues with nucleobases or by compensation of the negative charge of the rRNA backbone. These interactions are most often provided by regions of proteins without secondary structure elements, typically short clade-specific polypeptides in *Toxoplasma* and massively extended termini of conserved proteins in trypanosomes. Stabilization is possibly also aided by small molecules, such as a nucleotide triphosphate found associated with mtSSU rRNA in *Trypanosoma brucei* [7]. Another convergent feature of trypanosomal and apicomplexan mitochondrial rRNA is its posttranscriptional modification at 3′-ends (Fig 1B). All but 11 fragments of *Toxoplasma* rRNA are polyadenylated. The poly-A tails were proposed to provide handles, which facilitate the assembly and aid stability of the three-dimensional jigsaw puzzle [10]. Analogously, radially extended termini of mitochondrial rRNA fragments in alga *Polytomella magna* are stabilized by associated lineage-specific helical-repeat proteins [4]. Mature trypanosomal mitochondrial rRNAs contain 3′-polyuridine introduced by terminal uridylyl transferase RET1, but the role of the modification remains unclear [17,18].

Protein composition of mitoribosomes of the two parasitic protists also evolved in a convergent manner. On one hand, both groups lost several proteins of bacterial origin widespread in majority of other mitoribosomes, namely uL5m, bL34m, and uS7m. On the other hand, they gained the highest number of lineage-specific mitoribosomal proteins: 57 in trypanosomatids and 55 in *Toxoplasma* [7,9,10] (Fig 1B). For comparison, animal and plant mitoribosomes contain equally only 13 proteins not found in any other mitoribosomes [1,5]. It was proposed that newly acquired proteins replace lost rRNA elements in mitoribosomes [19]. However, comparisons of cryo-EM structures across eukaryotes indicate that reduced rRNA elements are functionally replaced by proteins only in a few cases, such as the loss of 5S rRNA discussed below. More commonly, newly gained proteins merely occupy spatial niches liberated by lost rRNA, particularly in lineages with a general tendency toward protein accretion, including the parasites discussed here. In other groups, rRNA loss appears to be largely uncompensated, as exemplified by green algae, whose mitoribosomes contain highly reduced rRNA with minimal recruitment of proteins in core regions [4].

**Fig 2:**
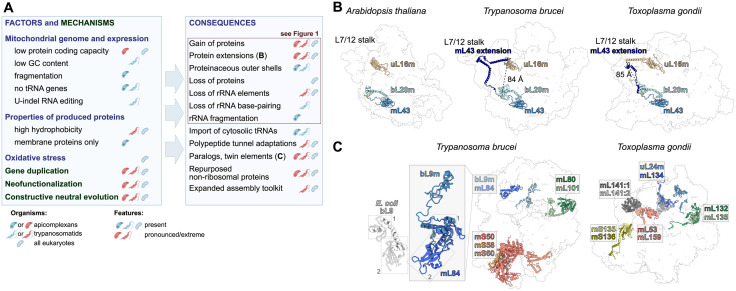
Evolution of mitochondrial ribosomes in apicomplexans, trypanosomatids, and eukaryotes in general. (A) Factors and mechanisms affecting evolution of mitochondrial ribosomes and their consequences. The pictograms indicate groups of organisms in which individual features occur (blue) or are pronounced (red). **(B)** Long convergent C-terminal extension of mL43 connects surface of mtLSU (shown as transparent surface) with the base of L7/12 stalk in apicomplexans and trypanosomatids. Ancestral-like state in *Arabidopsis thaliana* is shown for comparison. **(C) “**Twin elements” in the parasite’s mitoribosomes. Close-up view: *T. brucei* mL84 is a group-specific paralog of bL9m, a ribosomal protein of bacterial origin. While trypanosomal bL9m contains only the N-terminal domain (1) of bacterial bL9, the derived mL84 contains both the N-terminal and C-terminal (2) domains. Created by Ondřej Gahura using BioRender.com (https://biorender.com/shortURL) and licensed under CC BY 4.0.

Some of the lineage-specific proteins in trypanosomatids and apicomplexan are paralogs of various enzymes, transcription factors, or different kinds of other non-ribosomal proteins, documenting frequent contribution of neofunctionalization. Several couples of paralogs are found within mitoribosomal proteins themselves. In two cases, one in each group, a protein of bacterial origin has duplicated, and its modified copy got incorporated in the structure (uL24m and mL134 in *Toxoplasma* and bL9m and mL84 in trypanosomes; Fig 2). In other instances, two or three mitochondria- or lineage-specific paralogs co-occur in the ribosome, often in a form of heterodimer. These so called “twin elements” [10] represent another remarkable feature of mitoribosomes in both groups (Fig 2C).

A striking example of structural convergence is a C-terminal extension of mL43, one of mitoribosomal proteins without homologs in bacteria but recruited before the last eukaryotic common ancestor (LECA). mL43 contains a core with a thioredoxin fold and a C-terminal tail, which staples bL20m to the LSU by conserved interactions. The C-terminal tail is massively extended in trypanosomatids and apicomplexans. The extensions wind >80 Å to the conserved base of the L7/12-stalk, but their interactions with the L7/12 stalk base differ between the organisms (Fig 2B). Thus, the long-distance spanning extensions, which might facilitate communication between the surface and the key mobile protrusion involved in the translation cycle, are most likely products of convergent evolution.

## What drives or allows the increased complexity of the parasites’ mitoribosomes?

The substantial architectural sophistication of mitoribosomes in general and in the two parasitic groups specifically can stem from two non-mutually exclusive principles: (i) a relief of selection pressure due to largely decreased diversity of proteins produced in the organelle and (ii) emergence of new context that requires alterations to the status quo. The reduction of bacterial-like gene-rich genome of early protomitochondria to the state in extant eukaryotes with mitochondrial genomes encoding no more than about hundred proteins is associated with recruitment of new proteins to mitoribosomes (Fig 2A). Indeed, mitoribosomes inferred in LECA contained 26 proteins that are absent in alphaproteobacterial ribosomes [20] and were therefore gained after the endosymbiotic event, when the organellar genomes shrank substantially. However, reduction in protein-coding capacity *per se* is not sufficient to explain massive gain of ribosomal proteins. Genomes of plastids, the other endosymbiotic organelle, in land plants encode only 70–80 proteins, which is less than the inferred mitochondrial genome of LECA, yet possess ribosomes that have gained only four proteins during their evolution from a cyanobacterial ancestor.

It is reasonable to speculate that the changes of mitochondrial genomes in apicomplexans and trypanosomatids predetermined the unique architecture of their mitoribosomes (Fig 2A). Mitoribosomes in trypanosomatids contain two mitochondrially encoded proteins (uS3m and uS12m), whereas in apicomplexans—similar to mammals—all mitoribosomal proteins are encoded in the nuclear genome. Although the basis for the distinct patterns of mitoribosomal gene transfer in different eukaryotes is unclear, the absence of mitoribosomal proteins in the mitochondrial genome implies that regulation of stoichiometry between nuclear- and mitochondrially encoded proteins is no longer necessary. Mitochondrial genomes in both groups are devoid of any transfer RNAs (tRNAs). All tRNAs used in mitochondrial translation are encoded in nuclear genomes and are shared with cytosolic ribosomes [21,22]. Thus, the mitoribosomes derived from bacterial ribosomes accommodate the eukaryotic tRNAs. No specific adaptation is apparent from the structures, albeit it likely has occurred. Further, trypanosomatids and apicomplexans have independently lost 5S rRNA, and consequently contain purely proteinaceous central protuberance, a structural feature of mtLSU required for association with mtSSU. Remodeling of central protuberance is a common feature of mitoribosomes [3,23–25], and 5S rRNA has been lost multiple times in the evolution of eukaryotes [26]. Overall, any alterations in mitochondrial rRNA genes are directly mirrored in the rRNA structure. In apicomplexans, mitoribosomes got adapted to extensive fragmentation of mitochondrial DNA [27] and evolved mechanisms how to cope with massively fragmented rRNA [10]. In trypanosomatids, the overall low genomic GC content [28] contributed to the attenuation of scaffolding properties of originally double-stranded rRNA regions. As discussed above, both fragmentation and loss of intrinsically stable rRNA elements require co-evolution of stabilization, which necessitates acquisition of novel protein elements.

The need for rRNA stabilization, however, does not explain robust proteinaceous shells without essentially any RNA (Fig 1A). Mitochondria-specific proteinaceous elements were suggested to provide a protection of rRNA against increased oxidative stress in the organelle in mammals [29]. Accordingly, the massive outer shells can provide shielding against reactive oxygen species in the parasitic protists. However, it should be noted that in organisms such as green algae *C. reinhardtii* and *P. magna*, additional protein mass does not shield rRNA [4,6] and therefore the protective role cannot be the sole or universal explanation for extended outer layers. More plausibly, adoption of superficially located proteins was at least partially product of constructive neutral evolution, a mechanism wherein novel structural features are initially obtained without impact on fitness and only later are fixed by evolving interdependence with preexisting features [30]. Non-adaptive processes can be promoted by high mutation rates, and unusual organellar genomes or gene-expression pathways have been linked to elevated predicted mutation rates [31]. Yet apicomplexan and trypanosomatid genomes do not show exceptionally high rates [32,33], and these vary across species [34], leaving their contribution to mitoribosome evolution uncertain.

The expansion of the solvent-exposed side of the LSU has consequences for the polypeptide tunnel and its exit. In trypanosomes, the tunnel has been adapted to allow the passage of overall extremely hydrophobic nascent polypeptides. It was also proposed to branch into two exits, one for membrane proteins and the other for soluble proteins [7], although this conclusion was questioned based on the structure from *Leishmania major*, which indicated that the apparent branching occurs only until a late stage of assembly process but is absent in the mature mtLSU [8].

## Current research: How are the ribosomes pieced together?

As in macroscopic world, biogenesis of intricate molecular complexes requires toolkits. The tools are proteins termed assembly factors. In case of mitoribosomes, they facilitate RNA folding and modification, protein recruitment, or govern order of assembly events. Reduction and fragmentation of rRNA and increased number of proteins impose specific requirements for biogenesis of the mitoribosomes in trypanosomes and *Toxoplasma*. Presumably, the process depends on expanded repertoire of assembly factors. Indeed, structures of several mtLSU and mtSSU precursors revealed in total 68 assembly factors in *T. brucei* [8,35–39] (reviewed in [25]), outnumbering the biogenetic toolkit in well-studied mammalian systems, in which only up to 30 assembly factors have been identified [40–42]. Thus, at least a half of trypanosomal assembly factors is lineage-specific and likely co-evolved with the increased complexity of mitoribosomes. The new factors often emerge as products of gene duplication or expansion of protein families. The most striking example is mitochondrial initiation factor 3 (mt-IF3). The canonical function of mt-IF3 in translation initiation is extended in mammalian mitochondria to a role in mtSSU assembly. In trypanosomes, mt-IF3 gene has been duplicated. One paralog acts in translation initiation while the other, containing long extensions, is involved in the assembly [35,37].

The necessity to piece together multiple rRNA fragments along with gain of specific mitoribosomal proteins in *Toxoplasma* likely also requires engagement of additional assembly factors. Furthermore, the individual fragments must be present in stoichiometric quantities. Whether this is achieved on the transcription or post-transcription level needs to be elucidated. Nevertheless, additional factors orchestrating the stoichiometry are likely to be involved. To date, biogenesis of mitoribosomes in apicomplexans has not been studied experimentally. Homologs of several assembly factors of bacterial origin were identified in their genomes [43]. Some assembly mechanisms are thus shared with bacteria and mitoribosomes in other eukaryotes. *Trypanosoma brucei*, which in contrast to other organisms contains abundant pre-mitoribosomal complexes and therefore is a convenient model to study the assembly pathway by cryoEM, provided valuable insight into common principles of biogenesis of mitoribosomes [25,39]. Unlike relatively late stage of biogenesis, the early steps of mitoribosome biogenesis remain enigmatic and trypanosomes could be instrumental for their elucidation.

## Questions for future: How is mitochondrial protein synthesis in the parasites regulated?

Proteins encoded in mitochondrial genomes are predominantly essential components of complexes required for the function of the organelle, oxidative phosphorylation complexes, and mitoribosomes themselves. These complexes have dual genetic origin and their assembly, critical for maintenance of cellular homeostasis, requires coordinated gene expression of their nuclear and mitochondrial encoded components [44,45]. Regulatory mechanisms of mitochondrial translation, which control the expression of mitochondrial encoded proteins, have been studied in yeasts and mammals. Individual human mitochondrial mRNAs fold into distinct structures [46] and most fungal and some human mRNAs bind specific nuclear-encoded translation activators [47,48]. This controls interaction of mRNAs with mitoribosomes at the entry of the mRNA channel and translation initiation. mRNA structures also affect elongation rate to facilitate co-translational protein folding and insertion in the mitochondrial membrane [46] and mRNA-specific feedback loops enable translation only if the nascent product assembles into the respective complex. However, mRNA architecture, including presence or absence of UTRs, and translation activators, their function or their binding sites on mitoribosomes are typically phylogenetically restricted [47,49]. Unrelated eukaryotes, including trypanosomatids and apicomplexans, likely use different mechanisms or factors to regulate mitochondrial translation and orchestrate it with production of nuclear-encoded components. Apicomplexans can be suitable models to study mRNA-specific translation regulation, because their mitochondrial genomes encode only three proteins, components of complexes III and IV of electron transport chain [50]. If feedback between translation of the subunits and assembly of these complexes is essential in apicomplexans, as it is in mammals and yeast, it would support the “regulation” argument for the preservation of mitochondrial genomes in eukaryotes [51]. Mitochondrial translation is also affected by upstream stages of mitochondrial gene expression. In trypanosomatids, several mitochondrial transcripts undergo massive modification by site-specific insertion or deletion of up to hundreds of uridines [52]. Currently, it is unknown how mitoribosomes distinguish fully edited translation-competent mRNAs from unedited species or editing intermediates. Thus, regulation of mitochondrial translation and its interplay with upstream steps of gene expression remains to be studied in the parasites.
